# Synthesis and crystal structure of 4-benzyl-4-pentyl­morpholin-4-ium chloride

**DOI:** 10.1107/S2056989025006772

**Published:** 2025-08-05

**Authors:** Zarifa Yakhshilikova, Tursunali Kholikov, Sherzod Zhurakulov, Kambarali Turgunov

**Affiliations:** a National University of Uzbekistan named after Mirzo Ulugbek University Str 4 Tashkent 100174 Uzbekistan; b S. Yunusov Institute of the Chemistry of Plant Substances Academy of Sciences of Uzbekistan Mirzo Ulugbek Str 77 Tashkent 100170 Uzbekistan; c Turin Polytechnic University in Tashkent Kichik Khalka yuli str 17 100095 Tashkent Uzbekistan; University of Missouri-Columbia, USA

**Keywords:** crystal structure, mol­ecular structure, *N*-pentyl morpholine, benzyl chloride, quaternary morpholine halide

## Abstract

The reaction of *N*-pentyl­morpholine with benzyl chloride resulted in the title compound, C_16_H_26_ClNO, which crystallizes in the ortho­rhom­bic space group *Pna*2_1_ with *Z* = 4. In the crystal, the chloride ions are surrounded by four cations, forming layers.

## Abstract 2

An investigation is reported of the synthesis and crystal structure of 4-benzyl-4-pentyl­morpholin-4-ium chloride

## 1. Chemical context

Morpholine is a multipurpose chemical that is used as a solvent for resins, dyes and waxes. One of its most important uses is as a chemical inter­mediate in the preparation of pesticides (Muruganandam *et al.*, 2009 ▸). A number of morpholine derivatives have been described as analgesics and local anesthetics. The morpholino­methyl derivative of pyrizinamide (morphozinamide) has been found to be more effective in the treatment of tuberculosis than pyrizinamide (Sedavkina *et al.*, 1984 ▸). Quaternary morpholine halides were found to achieve total disinfection against *Staphylococcus aureus* ATCC 25923 and *Escherichia coli* ATCC 25922. (Morandini *et al.*, 2021 ▸). Additionally, most drugs containing a morpholine moiety in their structure have been found to exhibit significant biological properties (Basavaraja *et al.*, 2010 ▸). Quaternary morpholine halides are valuable precursors for the preparation of ionic liquids (ILs) by ion metathesis (Kim *et al.*, 2005 ▸). The excellent conductivity, broad electrochemical window, thermal stability, and low volatility of ILs have made them promising media for electrochemical processes (Zein El Abedin *et al.*, 2004 ▸, 2005 ▸). In particular, ILs based on the morpholinium cation are favored because of their low cost, easy synthesis, and electrochemical stability (Kim *et al.*, 2006 ▸). We report here a new example structure of this class.
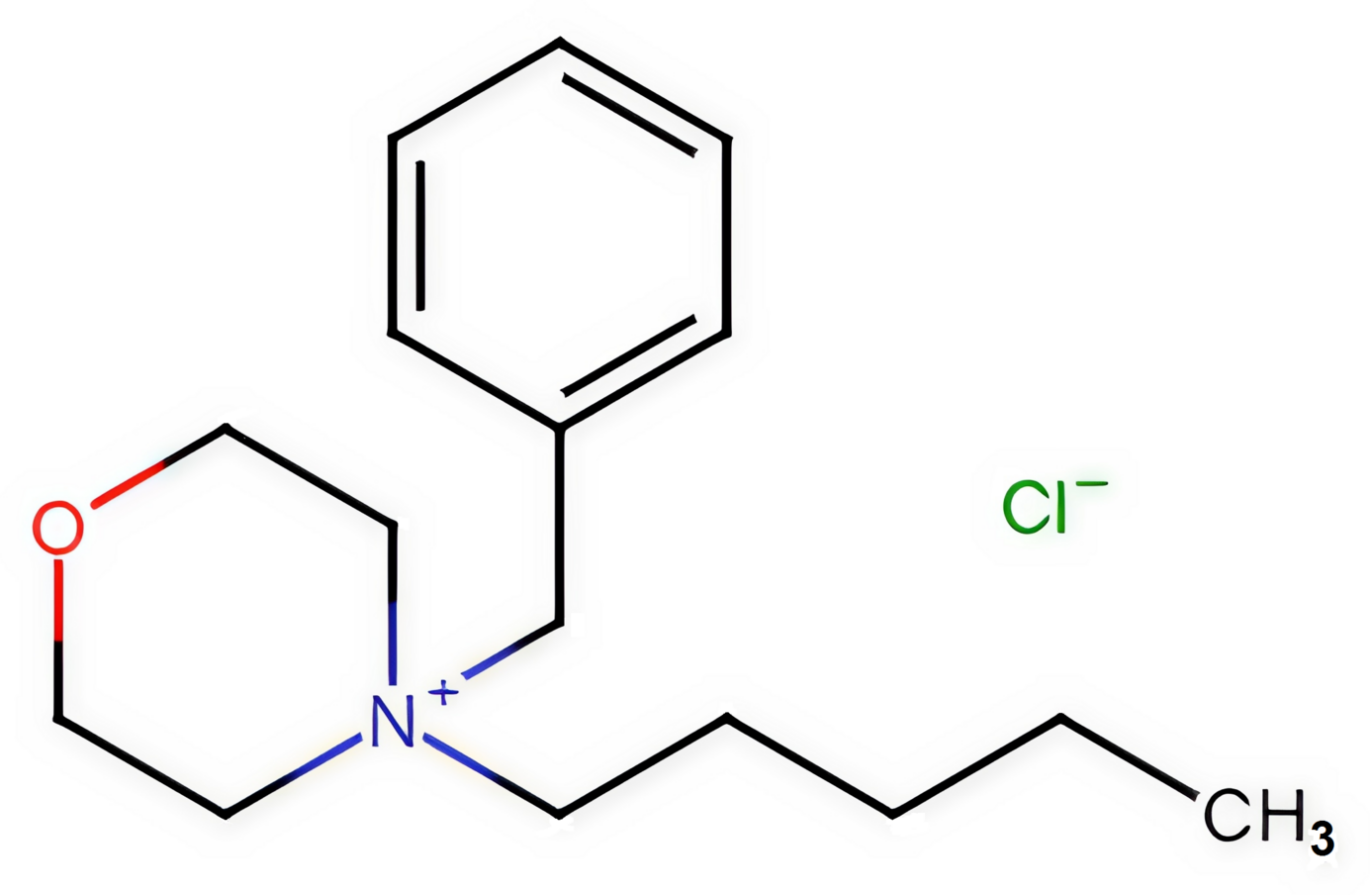


## 2. Structural commentary

**Figure 1:**
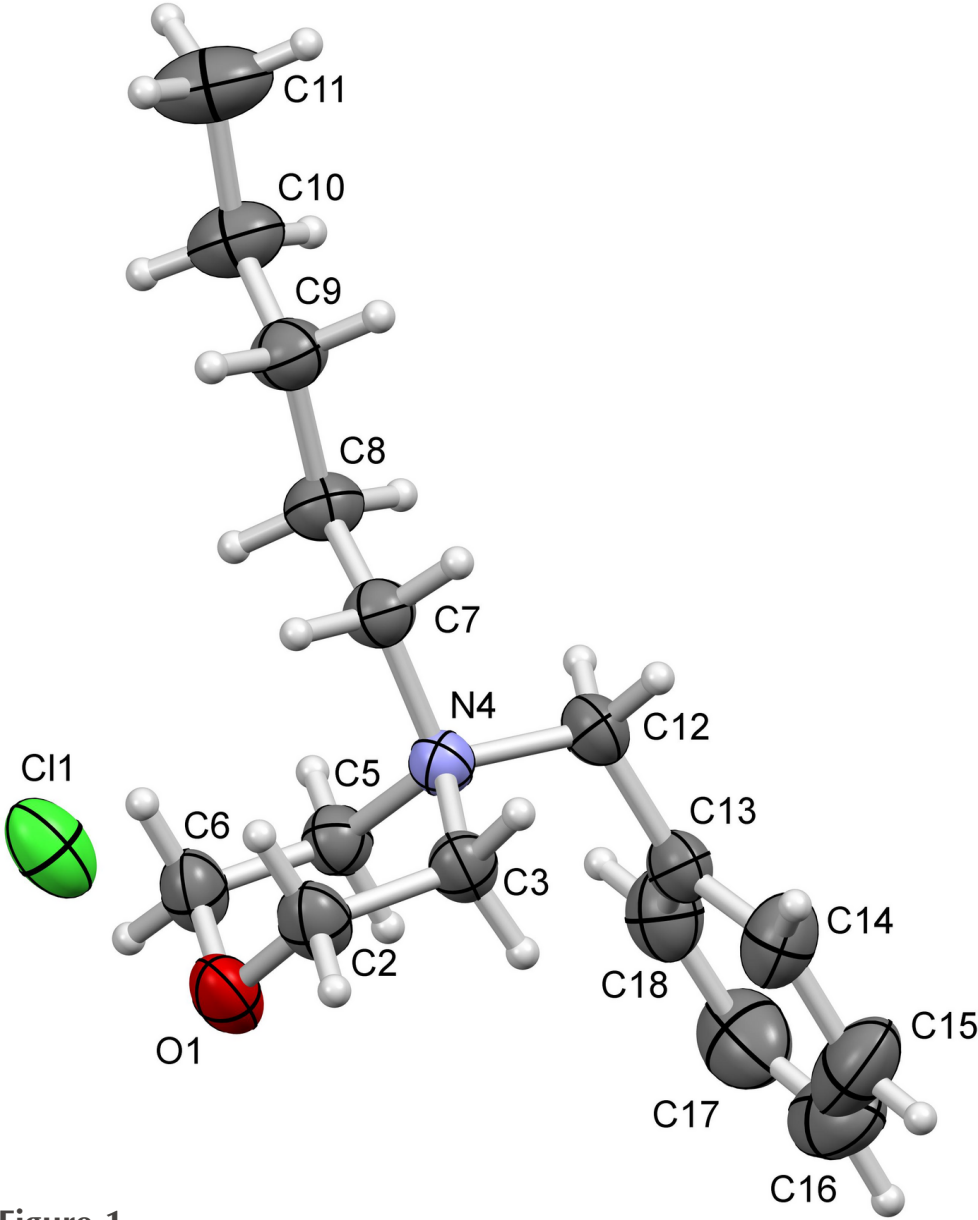
The mol­ecular structure of the title compound, with displacement ellipsoids drawn at the 50% probability level.

The title compound crystallizes in the ortho­rhom­bic space group *Pna*2_1_ with *Z* = 4. The asymmetric unit consists of a 4-benzyl-4-pentyl­morpholin-4-ium cation with a quaternary nitro­gen atom and the chloride counter-anion, which ensures neutrality (Fig. 1 ▸). The average C—N bond length of 1.521 Å and C—N—C angle of 109° are consistent with the geometry of a charged quaternary nitro­gen atom found in different structures (Rousselin & Clavel, 2024 ▸). In the cation, the morpholinium ring adopts a chair conformation with puckering parameters (Cremer & Pople, 1975 ▸) of the ring *Q* = 0.5711 (18) Å, θ = 4.26 (18)°, φ = 29 (2)°. Weak intra­molecular C—H ⋯Cl hydrogen bonds help to consolidate the conformation of the mol­ecule (Table 1 ▸). The pentyl group carbon atoms lie in a plane with an r.m.s. deviation of 0.0252 Å.

## 3. Supra­molecular features

**Figure 2:**
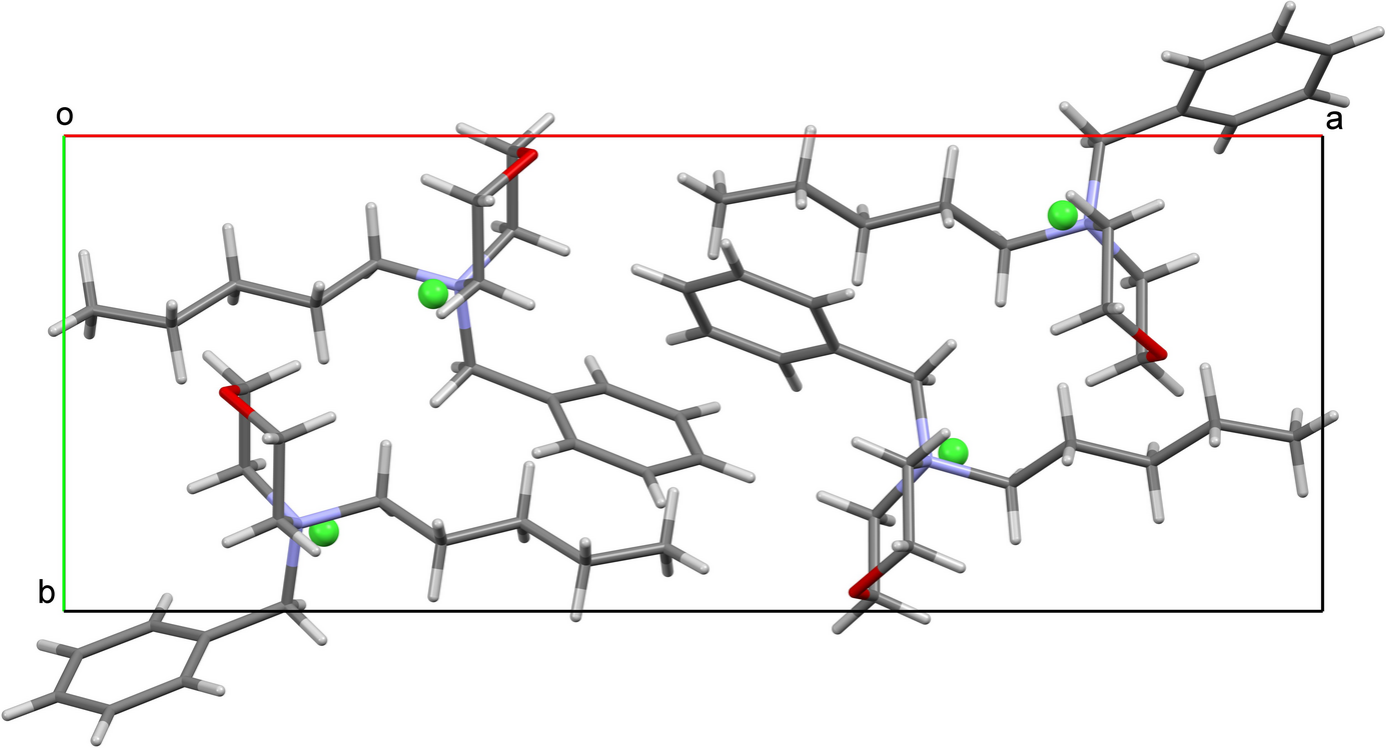
The packing of the title compound.

**Figure 3:**
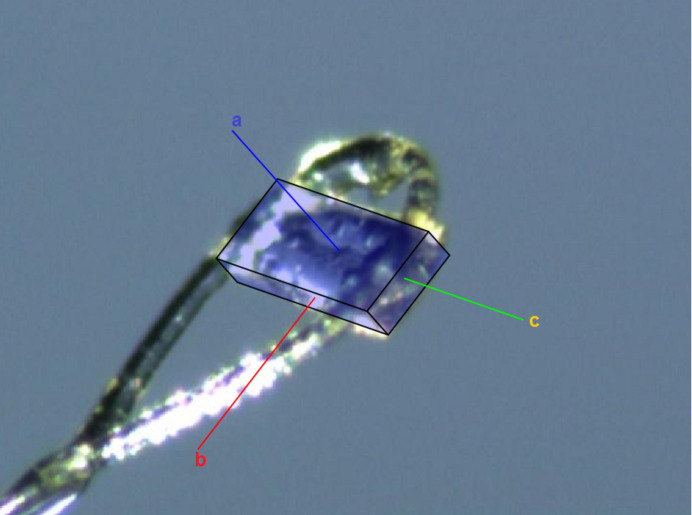
Screenshot from the face-indexing procedure (showing the unit-cell axes).

**Figure 4:**
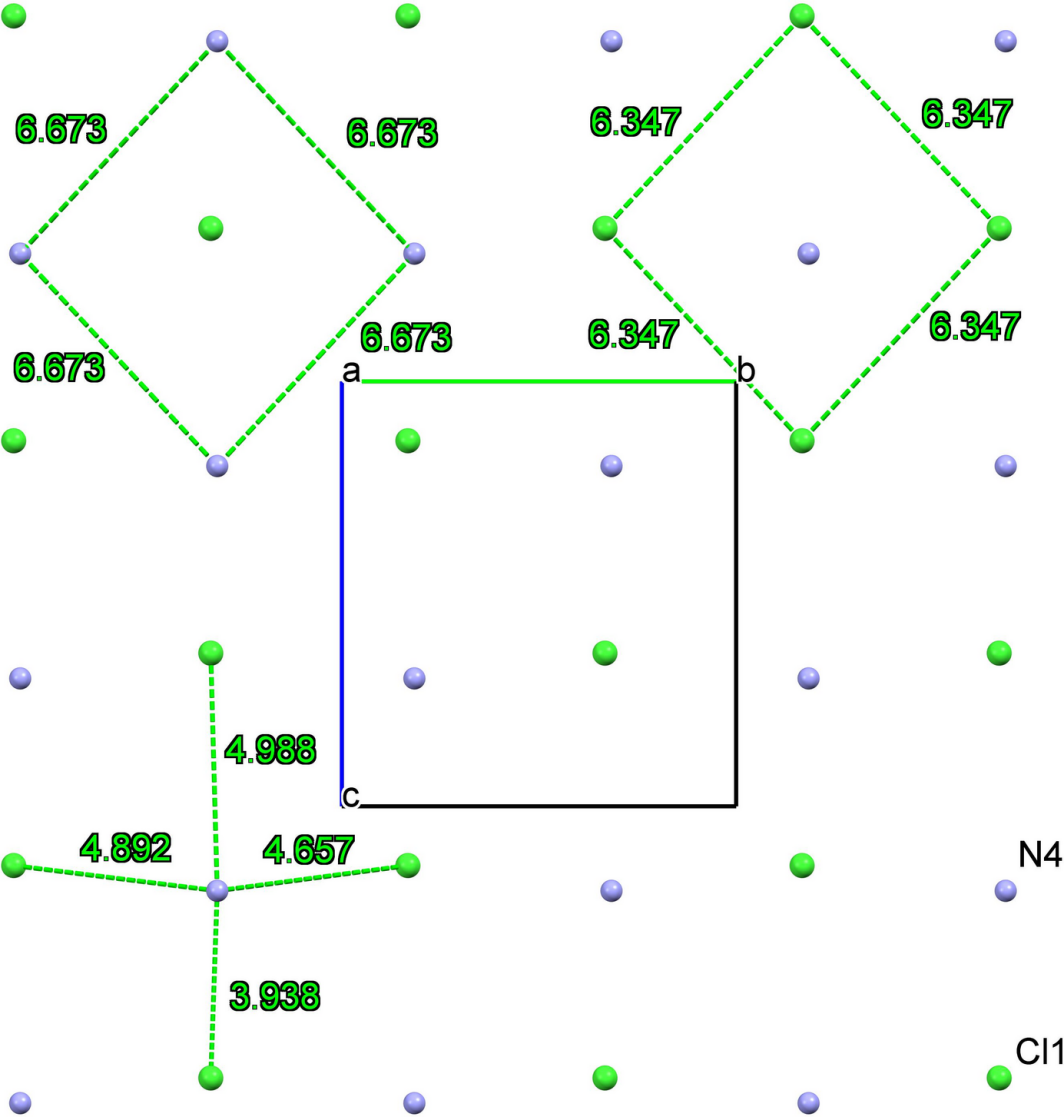
Distribution of positively charged nitro­gen atoms and chloride counter-ions in a layer. Interatomic distances are given in Å.

The crystal packing is shown in Fig. 2 ▸, where four cations, accompanied by counter-ions, are arranged head-to-tail in the unit cell. An examination of the distribution of the positively charged nitro­gen atoms in the morpholinium cations and the chloride counter-ions shows that the crystal forms ion layers parallel to the *bc* plane, which corresponds to the planar surface of the monocrystal (Fig. 3 ▸). Within these layers, each nitro­gen atom forms short contacts with four chloride ions at distances of 3.938 (2), 4.657 (2), 4.892 (2), and 4.988 (2) Å. The chloride ions are separated by a distance of 6.3470 (4) Å, forming a two-dimensional structure typical of salts with a cyclo­butane-like puckering conformation. Each chloride ion is surrounded by methyl­ene groups, which form weak C—H⋯Cl hydrogen bonds (Table 1 ▸). The arrangement and geometry of the nitro­gen atoms are similar, with a nitro­gen–nitro­gen distance of 6.673 (1) Å (Fig. 4 ▸). These layers are packed through the partial inter­calation of alkyl and phenyl groups along the *a* axis, forming C_ar_—H⋯π inter­actions (Table 1 ▸).

## 4. Database survey

A search of the Cambridge Structural Database (CSD, Version 5.46 of November 2024; Groom *et al.*, 2016 ▸) for structures containing a morpholine fragment with three bonded nitro­gen atom returned 2745 hits. A search for structures containing a morpholin-4-ium fragment returned 188 hits, while a search for the morpholin-4-ium fragment with a benzyl substituent produced 10 matches. Homologous structures with methyl and ethyl substituents are MOKJOM (Bian, 2009*a* ▸) and DOKYAE (Bian, 2009*b* ▸). The C—N bond length in a neutral morpholine fragment is approximately 1.46–1.48 Å (Groom *et al.*, 2016 ▸; Mutalliev *et al.*, 2022 ▸) while the C—N bond in a morpholin-4-ium structure, as mentioned above, measures around 1.52 Å.

## 5. Synthesis and crystallization

*N-pentyl morpholine*, *C_9_H_19_NO*. To a 50 ml round-bottom flask, 4.95 g (0.06 mol) of morpholine were added. After adding 0.6 mol of ethanol as the solvent, 0.06 mol of potassium carbonate (K_2_CO_3_) and then 7.50 ml (0.06 mol) of pentyl bromide were added. The reaction mixture was heated under reflux with magnetic stirring for 1–9 h (monitored by TLC). Afterward, the solvent was evaporated. The remaining potassium carbonate in the solution was dissolved in water, and the reaction product was extracted with chloro­form (CHCl_3_). After the chloro­form had evaporated, the residue was dried under vacuum. Yield 6.5 g (72.0%).

^1^ H-NMR (600 MHz, CDCl_3_, δ, ppm *J*/Hz): 0.85 (3H, *t*, *J* = 7.2, H-11), 1.26 (4H, *m*, H-9,10), 1.44 (2H, *kd*, *J* = 7.4, 2.1, H-8), 2.27 (2H, *dt*, *J* = 7.9, 2.4, H-7), 2.39 (4H, *s*, H-2,6), 3.68 (4H, *s*, H-3,5).

^13^C NMR (150 MHz, CDCl_3_, δ, ppm): 14.09 (C-11), 22.41 (C-10), 26.31 (C-8), 29.89 (C-9), 53.62 (C-2,6), 59.29 (C-7), 66,93 (C-3,5).

IR spectrum (KBr, ν_max_, cm^−1^): 2957, 2931, 2856, 2807, 1708, 1454, 1358, 1271, 1118, 1071-1757, 1034, 1004, 914, 864, 796, 628.

*4-Benzyl-4-pentyl­morpholin-4-ium chloride*, *C_16_H_26_ClNO*. To a 50 ml round-bottom flask, 2 g (0.013 mol) of *N*-pentyl morpholine were added. After adding 5.4 ml (0.104 mol) of aceto­nitrile as the solvent, 0.013 mol of potassium carbonate (K_2_CO_3_) were added, followed by benzyl chloride in a 1:1 ratio, *i.e*., 0.013 mol. The reaction mixture was heated under reflux with magnetic stirring for 5 h (monitored by TLC). Then the solvent was evaporated, the remaining potassium carbonate was dissolved in water, and the reaction product was extracted with chloro­form (CHCl_3_). After the chloro­form had evaporated, the product was dried under vacuum. The obtained product was purified using column chromatography. Yield 3.16 g (94.0%), m.p. 467–469 K. Single crystals were obtained by slow evaporation of an acetone solution.

^1^ H-NMR (600 MHz, CDCl_3_, δ, ppm *J*/Hz): 0.87 (3H, *t*, *J* = 6.19, H-11), 1.33 (4H, *m*, H-9,10), 1.80 (2H, *m*, H-8), 3.40 (2H, *dd*, *J* = 9.63, 2.38, H-7), 3.58 (4H, *m*, H-1,5), 3.76 (2H, *t*, *J* = 10.41 H-12), 3.95 (2H, *m*, H-2), 4.07 (2H, *d*, *J* = 13.94 H-4), 7.40 (3H, *m*, H-16,17,15), 7.58 (2H, *d*, *J* = 4.95 H-18,14).

^13^C NMR (150 MHz, CDCl_3_, δ, ppm): 13.94 (C-18), 21.84 (C-17), 22.31 (C-15), 28.42 (C-16), 56.33 (C-2,4), 56.98 (C-7), 60,62 (C-1,5), 64,85 (C-8), 126.77 (C-9), 129.42 (C-11,13), 130.86 (C-12), 133.40 (C-10,14).

IR spectrum (KBr, ν_max_, cm^−1^): 2976, 2951, 2873, 1495, 1458, 1393, 1216, 1121, 1050, 1019, 991, 946, 932, 912, 886, 860, 764.

## 6. Refinement

Crystal data, data collection and structure refinement details are summarized in Table 2 ▸. H atoms were placed in calculated positions and refined as riding on their parent atoms [C—H = 0.93–0.97 Å with *U*_iso_(H) = 1.2*U*_eq_(C)].

## Supplementary Material

Crystal structure: contains datablock(s) I, global. DOI: 10.1107/S2056989025006772/ev2018sup1.cif

Structure factors: contains datablock(s) I. DOI: 10.1107/S2056989025006772/ev2018Isup2.hkl

Supporting information file. DOI: 10.1107/S2056989025006772/ev2018Isup3.cml

CCDC reference: 2477070

Additional supporting information:  crystallographic information; 3D view; checkCIF report

## Figures and Tables

**Table 1:** Hydrogen-bond geometry (Å, °) *Cg*1 is the centroid of the benzene ring.

*D*—H⋯*A*	*D*—H	H⋯*A*	*D*⋯*A*	*D*—H⋯*A*
C2—H2*B*⋯Cl1^i^	0.97	2.78	3.708 (2)	160
C3—H3*B*⋯Cl1^ii^	0.97	2.78	3.645 (2)	149
C6—H6*B*⋯Cl1	0.97	2.77	3.477 (2)	130
C12—H12*B*⋯Cl1^ii^	0.97	2.75	3.639 (2)	153
C15—H15⋯*Cg*1^iii^	0.93	3.28	4.104 (3)	149

Symmetry codes: (i) 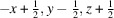; (ii) 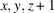; (iii) 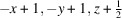.

**Table 2:** Experimental details

Crystal data	
Chemical formula	C_16_H_26_NO^+^·Cl^−^
*M*_r_	283.83
Crystal system, space group	Orthorhombic, *Pna*2_1_
Temperature (K)	293
*a*, *b*, *c* (Å)	21.8109 (4), 8.2459 (2), 8.8751 (2)
*V* (Å^3^)	1596.19 (6)
*Z*	4
Radiation type	Cu *K*α
μ (mm^−1^)	2.05
Crystal size (mm)	0.30 × 0.10 × 0.05

Data collection	
Diffractometer	Bruker D8 VENTURE dual wavelength Mo/Cu
Absorption correction	Multi-scan (*SADABS*; Krause *et al.*, 2015 ▸)
*T*_min_, *T*_max_	0.620, 0.754
No. of measured, independent and observed [*I* > 2σ(*I*)] reflections	22843, 3168, 3064
*R*_int_	0.034
(sin θ/λ)_max_ (Å^−1^)	0.625

Refinement	
*R*[*F*^2^ > 2σ(*F*^2^)], *wR*(*F*^2^), *S*	0.029, 0.074, 1.06
No. of reflections	3168
No. of parameters	173
No. of restraints	1
H-atom treatment	H-atom parameters constrained
Δρ_max_, Δρ_min_ (e Å^−3^)	0.16, −0.21
Absolute structure	Flack *x* determined using 1329 quotients [(*I*^+^)−(*I*^−^)]/[(*I*^+^)+(*I*^−^)] (Parsons *et al.*, 2013 ▸)
Absolute structure parameter	0.014 (6)

Computer programs: *APEX5* (Bruker, 2023 ▸), *SAINT* (Bruker, 2019 ▸), *SHELXT2018/2* (Sheldrick, 2015*a* ▸), *SHELXL2014/7* (Sheldrick, 2015*b* ▸), *Mercury* (Macrae *et al.*, 2020 ▸) and *publCIF* (Westrip, 2010 ▸).

## supplementary crystallographic information

### 4-Benzyl-4-pentylmorpholin-4-ium chloride Crystal data

**Table:**

C_16_H_26_NO^+^·Cl^−^	*D*_x_ = 1.181 Mg m^−^^3^
*M**_r_* = 283.83	Melting point: 468(2) K
Orthorhombic, *P**n**a*2_1_	Cu *K*α radiation, λ = 1.54178 Å
*a* = 21.8109 (4) Å	Cell parameters from 9944 reflections
*b* = 8.2459 (2) Å	θ = 4.1–74.5°
*c* = 8.8751 (2) Å	µ = 2.05 mm^−^^1^
*V* = 1596.19 (6) Å^3^	*T* = 293 K
*Z* = 4	Plate, colourless
*F*(000) = 616	0.30 × 0.10 × 0.05 mm

### 4-Benzyl-4-pentylmorpholin-4-ium chloride Data collection

**Table:**

Bruker D8 VENTURE dual wavelength Mo/Cu diffractometer	3168 independent reflections
Radiation source: microfocus X-ray source, Incoatec IµS 3.0 Microfocus Source	3064 reflections with *I* > 2σ(*I*)
Mirror monochromator	*R*_int_ = 0.034
ω–φ scans	θ_max_ = 74.6°, θ_min_ = 4.1°
Absorption correction: multi-scan (SADABS; Krause *et al.*, 2015)	*h* = −27→25
*T*_min_ = 0.620, *T*_max_ = 0.754	*k* = −9→10
22843 measured reflections	*l* = −11→10

### 4-Benzyl-4-pentylmorpholin-4-ium chloride Refinement

**Table:**

Refinement on *F*^2^	Hydrogen site location: inferred from neighbouring sites
Least-squares matrix: full	H-atom parameters constrained
*R*[*F*^2^ > 2σ(*F*^2^)] = 0.029	*w* = 1/[σ^2^(*F*_o_^2^) + (0.039*P*)^2^ + 0.1423*P*] where *P* = (*F*_o_^2^ + 2*F*_c_^2^)/3
*wR*(*F*^2^) = 0.074	(Δ/σ)_max_ < 0.001
*S* = 1.06	Δρ_max_ = 0.16 e Å^−^^3^
3168 reflections	Δρ_min_ = −0.21 e Å^−^^3^
173 parameters	Absolute structure: Flack *x* determined using 1329 quotients [(*I*^+^)-(*I*^-^)]/[(*I*^+^)+(*I*^-^)] (Parsons *et al.*, 2013)
1 restraint	Absolute structure parameter: 0.014 (6)

### 4-Benzyl-4-pentylmorpholin-4-ium chloride Special details

**Table:**

Geometry. All esds (except the esd in the dihedral angle between two l.s. planes) are estimated using the full covariance matrix. The cell esds are taken into account individually in the estimation of esds in distances, angles and torsion angles; correlations between esds in cell parameters are only used when they are defined by crystal symmetry. An approximate (isotropic) treatment of cell esds is used for estimating esds involving l.s. planes.

### 4-Benzyl-4-pentylmorpholin-4-ium chloride Fractional atomic coordinates and isotropic or equivalent isotropic displacement parameters (Å^2^)

**Table:**

	*x*	*y*	*z*	*U*_iso_*/*U*_eq_	
Cl1	0.29346 (3)	0.33262 (7)	0.13961 (7)	0.06703 (18)	
O1	0.37213 (7)	0.03995 (18)	0.55365 (16)	0.0541 (4)	
N4	0.31419 (6)	0.31617 (17)	0.69908 (18)	0.0378 (3)	
C2	0.35629 (9)	0.0320 (2)	0.7090 (2)	0.0489 (4)	
H2A	0.3854	−0.0368	0.7614	0.059*	
H2B	0.3159	−0.0160	0.7195	0.059*	
C3	0.35639 (9)	0.1990 (2)	0.7799 (2)	0.0431 (4)	
H3A	0.3978	0.2418	0.7786	0.052*	
H3B	0.3437	0.1900	0.8843	0.052*	
C5	0.32960 (9)	0.3091 (2)	0.5339 (2)	0.0421 (4)	
H5A	0.3700	0.3553	0.5177	0.050*	
H5B	0.3002	0.3736	0.4778	0.050*	
C6	0.32864 (10)	0.1372 (2)	0.4759 (2)	0.0492 (4)	
H6A	0.2880	0.0919	0.4892	0.059*	
H6B	0.3380	0.1369	0.3690	0.059*	
C7	0.24799 (8)	0.2672 (2)	0.7317 (2)	0.0428 (4)	
H7A	0.2441	0.1512	0.7163	0.051*	
H7B	0.2394	0.2890	0.8370	0.051*	
C8	0.19981 (8)	0.3526 (2)	0.6362 (3)	0.0468 (4)	
H8A	0.2029	0.3170	0.5323	0.056*	
H8B	0.2064	0.4689	0.6391	0.056*	
C9	0.13639 (8)	0.3120 (2)	0.6983 (3)	0.0502 (5)	
H9A	0.1303	0.1956	0.6940	0.060*	
H9B	0.1343	0.3447	0.8032	0.060*	
C10	0.08545 (9)	0.3951 (3)	0.6118 (3)	0.0613 (6)	
H10A	0.0858	0.3565	0.5085	0.074*	
H10B	0.0933	0.5109	0.6098	0.074*	
C11	0.02260 (11)	0.3655 (4)	0.6789 (4)	0.0841 (10)	
H11A	−0.0082	0.4107	0.6139	0.126*	
H11B	0.0202	0.4160	0.7762	0.126*	
H11C	0.0159	0.2509	0.6892	0.126*	
C12	0.32319 (8)	0.4877 (2)	0.7628 (2)	0.0453 (4)	
H12A	0.2952	0.5606	0.7114	0.054*	
H12B	0.3120	0.4868	0.8685	0.054*	
C13	0.38755 (8)	0.5547 (2)	0.7483 (2)	0.0437 (4)	
C14	0.42946 (11)	0.5302 (3)	0.8635 (3)	0.0612 (6)	
H14	0.4180	0.4735	0.9496	0.073*	
C15	0.48864 (12)	0.5907 (3)	0.8500 (4)	0.0801 (8)	
H15	0.5172	0.5711	0.9256	0.096*	
C16	0.50513 (13)	0.6793 (3)	0.7253 (5)	0.0831 (9)	
H16	0.5449	0.7188	0.7161	0.100*	
C17	0.46324 (14)	0.7091 (3)	0.6154 (4)	0.0769 (8)	
H17	0.4742	0.7719	0.5326	0.092*	
C18	0.40444 (11)	0.6468 (2)	0.6257 (3)	0.0584 (5)	
H18	0.3762	0.6673	0.5495	0.070*	

### 4-Benzyl-4-pentylmorpholin-4-ium chloride Atomic displacement parameters (Å^2^)

**Table:**

	*U*^11^	*U*^22^	*U*^33^	*U*^12^	*U*^13^	*U*^23^
Cl1	0.0981 (4)	0.0586 (3)	0.0444 (2)	0.0226 (3)	0.0031 (3)	0.0032 (3)
O1	0.0621 (9)	0.0502 (8)	0.0500 (8)	0.0156 (6)	−0.0040 (7)	−0.0115 (6)
N4	0.0375 (7)	0.0369 (7)	0.0389 (8)	0.0034 (5)	−0.0024 (6)	−0.0031 (6)
C2	0.0556 (11)	0.0426 (10)	0.0483 (11)	0.0106 (8)	−0.0070 (9)	−0.0021 (9)
C3	0.0452 (9)	0.0433 (9)	0.0409 (9)	0.0078 (7)	−0.0066 (8)	−0.0025 (8)
C5	0.0435 (9)	0.0452 (10)	0.0376 (9)	0.0017 (7)	−0.0001 (7)	−0.0011 (7)
C6	0.0596 (12)	0.0470 (10)	0.0409 (10)	0.0041 (8)	−0.0070 (9)	−0.0069 (8)
C7	0.0385 (8)	0.0427 (9)	0.0473 (9)	0.0002 (7)	0.0003 (7)	0.0010 (8)
C8	0.0396 (9)	0.0508 (9)	0.0501 (9)	0.0027 (7)	−0.0008 (9)	0.0026 (10)
C9	0.0419 (9)	0.0503 (10)	0.0583 (12)	−0.0014 (8)	0.0010 (9)	−0.0007 (9)
C10	0.0434 (10)	0.0626 (12)	0.0780 (17)	0.0017 (9)	0.0005 (10)	0.0120 (11)
C11	0.0460 (12)	0.0957 (19)	0.110 (3)	0.0070 (11)	0.0087 (13)	0.0243 (17)
C12	0.0468 (9)	0.0386 (9)	0.0505 (11)	0.0034 (7)	−0.0001 (8)	−0.0088 (8)
C13	0.0460 (9)	0.0341 (8)	0.0510 (10)	0.0011 (7)	0.0000 (8)	−0.0075 (7)
C14	0.0660 (13)	0.0534 (12)	0.0642 (13)	−0.0100 (10)	−0.0163 (12)	0.0010 (10)
C15	0.0621 (14)	0.0643 (15)	0.114 (2)	−0.0117 (12)	−0.0296 (15)	−0.0005 (16)
C16	0.0594 (13)	0.0580 (14)	0.132 (3)	−0.0143 (11)	0.0110 (17)	−0.0100 (16)
C17	0.0891 (18)	0.0552 (13)	0.086 (2)	−0.0174 (12)	0.0222 (16)	0.0003 (14)
C18	0.0734 (14)	0.0413 (9)	0.0604 (13)	−0.0024 (9)	−0.0029 (12)	0.0001 (11)

### 4-Benzyl-4-pentylmorpholin-4-ium chloride Geometric parameters (Å, º)

**Table:**

O1—C6	1.421 (3)	C9—H9A	0.9700
O1—C2	1.423 (2)	C9—H9B	0.9700
N4—C5	1.506 (3)	C10—C11	1.515 (3)
N4—C3	1.515 (2)	C10—H10A	0.9700
N4—C7	1.527 (2)	C10—H10B	0.9700
N4—C12	1.536 (2)	C11—H11A	0.9600
C2—C3	1.514 (3)	C11—H11B	0.9600
C2—H2A	0.9700	C11—H11C	0.9600
C2—H2B	0.9700	C12—C13	1.514 (3)
C3—H3A	0.9700	C12—H12A	0.9700
C3—H3B	0.9700	C12—H12B	0.9700
C5—C6	1.508 (3)	C13—C18	1.378 (3)
C5—H5A	0.9700	C13—C14	1.386 (3)
C5—H5B	0.9700	C14—C15	1.389 (3)
C6—H6A	0.9700	C14—H14	0.9300
C6—H6B	0.9700	C15—C16	1.374 (5)
C7—C8	1.522 (3)	C15—H15	0.9300
C7—H7A	0.9700	C16—C17	1.359 (5)
C7—H7B	0.9700	C16—H16	0.9300
C8—C9	1.526 (3)	C17—C18	1.384 (4)
C8—H8A	0.9700	C17—H17	0.9300
C8—H8B	0.9700	C18—H18	0.9300
C9—C10	1.514 (3)		
			
C6—O1—C2	109.55 (15)	C10—C9—C8	112.49 (18)
C5—N4—C3	107.53 (14)	C10—C9—H9A	109.1
C5—N4—C7	112.67 (14)	C8—C9—H9A	109.1
C3—N4—C7	108.43 (14)	C10—C9—H9B	109.1
C5—N4—C12	111.44 (14)	C8—C9—H9B	109.1
C3—N4—C12	109.60 (13)	H9A—C9—H9B	107.8
C7—N4—C12	107.12 (13)	C9—C10—C11	113.1 (2)
O1—C2—C3	111.15 (17)	C9—C10—H10A	109.0
O1—C2—H2A	109.4	C11—C10—H10A	109.0
C3—C2—H2A	109.4	C9—C10—H10B	109.0
O1—C2—H2B	109.4	C11—C10—H10B	109.0
C3—C2—H2B	109.4	H10A—C10—H10B	107.8
H2A—C2—H2B	108.0	C10—C11—H11A	109.5
C2—C3—N4	112.47 (14)	C10—C11—H11B	109.5
C2—C3—H3A	109.1	H11A—C11—H11B	109.5
N4—C3—H3A	109.1	C10—C11—H11C	109.5
C2—C3—H3B	109.1	H11A—C11—H11C	109.5
N4—C3—H3B	109.1	H11B—C11—H11C	109.5
H3A—C3—H3B	107.8	C13—C12—N4	115.05 (14)
N4—C5—C6	111.45 (16)	C13—C12—H12A	108.5
N4—C5—H5A	109.3	N4—C12—H12A	108.5
C6—C5—H5A	109.3	C13—C12—H12B	108.5
N4—C5—H5B	109.3	N4—C12—H12B	108.5
C6—C5—H5B	109.3	H12A—C12—H12B	107.5
H5A—C5—H5B	108.0	C18—C13—C14	119.1 (2)
O1—C6—C5	110.81 (16)	C18—C13—C12	121.06 (19)
O1—C6—H6A	109.5	C14—C13—C12	119.7 (2)
C5—C6—H6A	109.5	C13—C14—C15	119.8 (3)
O1—C6—H6B	109.5	C13—C14—H14	120.1
C5—C6—H6B	109.5	C15—C14—H14	120.1
H6A—C6—H6B	108.1	C16—C15—C14	120.2 (3)
C8—C7—N4	115.16 (16)	C16—C15—H15	119.9
C8—C7—H7A	108.5	C14—C15—H15	119.9
N4—C7—H7A	108.5	C17—C16—C15	119.9 (2)
C8—C7—H7B	108.5	C17—C16—H16	120.1
N4—C7—H7B	108.5	C15—C16—H16	120.1
H7A—C7—H7B	107.5	C16—C17—C18	120.6 (3)
C7—C8—C9	108.86 (19)	C16—C17—H17	119.7
C7—C8—H8A	109.9	C18—C17—H17	119.7
C9—C8—H8A	109.9	C13—C18—C17	120.3 (2)
C7—C8—H8B	109.9	C13—C18—H18	119.9
C9—C8—H8B	109.9	C17—C18—H18	119.9
H8A—C8—H8B	108.3		
			
C6—O1—C2—C3	−60.6 (2)	C8—C9—C10—C11	−175.9 (2)
O1—C2—C3—N4	56.3 (2)	C5—N4—C12—C13	−59.3 (2)
C5—N4—C3—C2	−51.0 (2)	C3—N4—C12—C13	59.6 (2)
C7—N4—C3—C2	71.1 (2)	C7—N4—C12—C13	176.99 (16)
C12—N4—C3—C2	−172.25 (16)	N4—C12—C13—C18	92.4 (2)
C3—N4—C5—C6	52.57 (19)	N4—C12—C13—C14	−90.8 (2)
C7—N4—C5—C6	−66.85 (19)	C18—C13—C14—C15	−3.6 (3)
C12—N4—C5—C6	172.70 (15)	C12—C13—C14—C15	179.6 (2)
C2—O1—C6—C5	62.8 (2)	C13—C14—C15—C16	2.2 (4)
N4—C5—C6—O1	−60.2 (2)	C14—C15—C16—C17	0.6 (4)
C5—N4—C7—C8	−50.2 (2)	C15—C16—C17—C18	−2.0 (4)
C3—N4—C7—C8	−169.13 (16)	C14—C13—C18—C17	2.3 (3)
C12—N4—C7—C8	72.7 (2)	C12—C13—C18—C17	179.0 (2)
N4—C7—C8—C9	−171.03 (16)	C16—C17—C18—C13	0.5 (4)
C7—C8—C9—C10	178.75 (19)		

### 4-Benzyl-4-pentylmorpholin-4-ium chloride Hydrogen-bond geometry (Å, º)

*Cg*1 is the centroid of the benzene ring.

**Table:**

*D*—H···*A*	*D*—H	H···*A*	*D*···*A*	*D*—H···*A*
C2—H2*B*···Cl1^i^	0.97	2.78	3.708 (2)	160
C3—H3*B*···Cl1^ii^	0.97	2.78	3.645 (2)	149
C6—H6*B*···Cl1	0.97	2.77	3.477 (2)	130
C12—H12*B*···Cl1^ii^	0.97	2.75	3.639 (2)	153
C15—H15···*Cg*1^iii^	0.93	3.28	4.104 (3)	149

Symmetry codes: (i) −*x*+1/2, *y*−1/2, *z*+1/2; (ii) *x*, *y*, *z*+1; (iii) −*x*+1, −*y*+1, *z*+1/2.
